# Compound Heterozygous Null Variants in *ITGB4* Gene Causing Severe Phenotype of Junctional Epidermolysis Bullosa With Pyloric Atresia in Thai Newborn: Genotype–Phenotype Correlation From a Case Report and Review of the Literature

**DOI:** 10.1155/ijog/2680052

**Published:** 2025-09-30

**Authors:** Jeerawan Klangjorhor, Mallika Pomrop, Patcharawadee Thongkumkoon, Nonthanan Moonsan, Ratchadaporn Khiaomai, Arnat Pasena, Pathacha Suksakit, Chulabhorn Pruksachatkun, Maliwan Tengsujaritkul

**Affiliations:** ^1^ Center of Multidisciplinary Technology for Advanced Medicine (CMUTEAM), Faculty of Medicine, Chiang Mai University, Chiang Mai, Thailand, cmu.ac.th; ^2^ Office of Research Administration, Chiang Mai University, Chiang Mai, Thailand, cmu.ac.th; ^3^ Department of Pediatrics, Faculty of Medicine, Chiang Mai University, Chiang Mai, Thailand, cmu.ac.th

**Keywords:** epidermolysis bullosa, junctional epidermolysis bullosa, pyloric atresia, Thai, whole exome sequencing

## Abstract

Epidermolysis bullosa (EB) is a genetically heterogeneous skin fragility disorder. Some subtypes also involve other organs, including the pulmonary, gastrointestinal, and renal systems. One severe form, junctional epidermolysis bullosa (JEB), is characterized by cleavage within the skin layers. The rarest and most lethal spectrum of this disorder includes pyloric atresia (PA), known as JEB‐PA. The number of reported cases in Thailand is limited. Using trio whole exome sequencing, we identified a Thai newborn with lethal JEB‐PA caused by novel null compound heterozygous variants in *ITGB4*. The patient carries a known nonsense variant (c.2533C>T) and a novel frameshift indel variant (c.1614delT) in *ITGB4*, inherited from her parents. Both are null variants that result in a premature termination codon (PTC). A comprehensive review of existing literature was conducted to gather information on disease‐causing variants and genotype–phenotype correlations in JEB‐PA. We summarized 50 previously reported JEB‐PA cases, detailing disease severity and *ITGB4* variants. We found that nonsense and frameshift indels were predominant in lethal cases (32/62 alleles), whereas missense variants were more common in nonlethal forms (18/38 alleles). Pathogenic variants were most frequently located in the Fibronectin II–like domain (FnIII) and cytoplasmic domain (CP) for null variants and in the von Willebrand factor Type A (VWFA) domain for missense variants. We emphasize the importance of genetic testing in these heterogeneous skin disorders. Molecular results reveal the disorder’s diagnosis, provide a precise prognosis, and guide the genetic counseling process for the family. Moreover, understanding pathomolecular mechanisms can lead to potential future treatments.

## 1. Introduction

Epidermolysis bullosa (EB) is a group of rare inherited genodermatoses characterized by skin fragility and blistering. Although EB is classified as a monogenic disorder, pathogenic variants in 16 different protein‐coding genes expressed in the skin have been identified as causative [1, 2]. Therefore, EB exhibits genetic heterogeneity, leading to various clinical features and severity. The most up‐to‐date EB classification is based on the skin layers where blisters originate. There are four main types, including EB simplex (intraepidermal layer), junctional epidermolysis bullosa (JEB) (within the lamina lucida of the basement membrane layer), dystrophic EB (below the basement membrane or layer of sublaminar densa), and Kindler EB (mixed skin layer cleavage pattern) [1, 2]. Furthermore, there are a total of 34 distinct subtypes of EB presenting a wide range of phenotypes [2]. The genotype–phenotype correlation depends not only on the specific gene but also on the type of variants [1]. Biallelic null variants are typically found in severe cases with early lethality, while missense variants are involved in milder forms. EB is a group of inherited skin disorders that primarily affect the epidermis and/or dermis, depending on the subtype, and can also involve mucous membranes of various internal organs such as the respiratory, gastrointestinal, and urinary tracts.

JEB is one of the severe and very rare forms of this spectrum, characterized by cleavage in the lamina lucida of basement membrane. The manifestation of this condition varies from an intermediate form with later onset to a severe form with early lethality [2]. Some subtypes of JEB are associated with internal organ involvement, such as pyloric atresia, interstitial lung disease, and nephrotic syndrome. Other manifestations previously reported include alopecia, enamel defects, and dystrophy or the absence of nails. Several genes have previously been reported as the cause of JEB, with a total of seven genes identified so far. These include *LAMA3*, *LAMB3*, *LMAC2*, which encode laminin 332, *COL17A1* encoding collagen XVII, *ITGB4* ‐and *ITGA6* encoding *α*6*β*4 integrin, and *ITGA3* encoding integrin *α*3 subunit, resulting in distinct phenotypes of a total of nine subtypes [2]. All genes are associated with an autosomal recessive (AR) inheritance pattern [1, 3]. However, although the incidence of JEB is relatively low, several cases with autosomal dominant (AD) inheritance have been reported [4, 5], indicating that both inheritance patterns may occur depending on the specific gene variant. Alternatively, other undisclosed modifying genetic or epigenetic factors may also play a role [4]. One of the rarest subtypes of JEB, the form co‐occurring with pyloric atresia (JEB‐PA; JEB5B; OMIM #226730), is associated with severe phenotypes, including pyloric atresia or stenosis, congenital absence of skin, and early lethality. This subtype results from alterations in *ITGB4*. Another subtype which has the same clinical presentation but less commonly is from *ITGA6* (JEB6; OMIM #619817). The varying levels of mutation in *ITGB4* result in different clinical manifestations as well.

In Thailand, there are a limited number of case reports on EB, and only a few cases have undergone mutation analysis. Most diagnoses have relied on histological examination of skin biopsies. There have been only five reported cases of JEB, with two of them being JEB‐PA [6, 7]. Only one case has been identified with an *ITGB4* pathogenic null variant using whole exome sequencing [6]. Here, we report a case of a newborn with severe JEB‐PA and detected biallelic null variants in the *ITGB4* gene as the cause of the disease. Moreover, we reviewed the literature and discussed disease‐causing variants and the genotype–phenotype correlation in JEB‐PA cases.

## 2. Materials and Methods

### 2.1. Ethical Approval Statement

Written informed consent was obtained from the patient’s parents. This study was approved by the Research Ethics Committee of the Faculty of Medicine, Chiang Mai University (Study Code: PED‐2564‐08528), and conducted in accordance with the Declaration of Helsinki.

### 2.2. Whole Exome Sequencing and Data Analysis

The genomic DNA was extracted from PBMC using the chloroform/isoamyl alcohol method. The quantity and integrity of the DNA were determined using NanoDrop and 1.5% agarose gel electrophoresis, respectively. Whole exome sequencing was performed on an Illumina NovaSeq 6000 by Macrogen (Seoul, Republic of Korea) using Agilent SureSelect V7 and 12G for exome capture, with 150‐bp paired‐end reads and a coverage depth of 200×. The FASTQ file was checked for quality with FastQC software Version 0.11.3 [8]. Adapters and primers were removed from the raw read sequences using Cutadapt software Version 3.1 [9]. The cleaned reads were mapped to the human reference genome (hg19) using BWA‐MEM software Version 0.7.10 [10]. Variant calling files (VCFs) were generated using the HaplotypeCaller GATK Best Practices (https://gatk.broadinstitute.org/hc/en-us) with default parameters.

The variant prioritization and interpretation were done as a trio analysis. The variants were filtered using gene lists associated with patient phenotypes (HPO term: aplasia cutis congenita HP:0001057), including *ALG9*, *ALX4*, *ALX4*, *ARHGAP31*, *ATP6V1B2 BMS1*, *COL17A1*, *COL7A1*, *COX7B*, *CPLX1*, *CTBP1*, *DLL4*, *DOCK6*, *DSP*, *EOGT*, *FGFRL1*, *HSPA9*, *ITGA6*, *ITGB4*, *KCTD1*, *KRAS*, *KRT14*, *KRT5*, *LAMA3*, *AMB3*, *LAMC2*, *LETM1*, *MCTP2*, *MMP1*, *MSX2*, *NELFA*, *NOTCH1*, *NSD2*, *PIGG*, *PLEC*, *RBPJ*, *TAF1*, *TFAP2A*, *UBA2*, and *UBR1*. Variants with a minor allele frequency (MAF) greater than 0.05, according to the Genome Aggregation Database (gnomAD, https://gnomad.broadinstitute.org/) and the Thai Reference Exome (T‐REx) database (https://trex.nbt.or.th/), were filtered out. The pathogenicity of missense variants was predicted using computational tools such as REVEL, BayesDel, and M‐CAP. The pathogenicity of candidate variants was classified according to the standards and guidelines of the American College of Medical Genetics and Genomics and the Association of Molecular Pathology (ACMG/AMP), as well as the updated additional guidelines from ClinGen (https://www.clinicalgenome.org/).

### 2.3. Sanger Sequencing

The variant validation and segregation were performed by Sanger sequencing. Primers were designed using Primer‐BLAST software (https://www.ncbi.nlm.nih.gov/tools/primer-blast/). The primer sequences are as follows: *ITGB4*‐Exon13‐F: 5 ^′^‐CTGTTTCGGGGAATGACCAGTT‐3 ^′^; *ITGB4*‐Exon13‐R: 5 ^′^‐AGAGAACCTCAGGGGAGTTGGA‐3 ^′^; *ITGB4*‐Exon21‐F: 5 ^′^‐CCAGGCATCCCCTGATCCTA‐3 ^′^; and *ITGB4*‐Exon21‐R: 5 ^′^‐GAGACCCCTCTCCCCTAGTT‐3 ^′^.

### 2.4. Genotype–Phenotype Correlation Analysis

We have identified cases with JEB‐PA, including 1 case from our study and 49 cases from the literature, which reported clinical data along with *ITGB4* variants. We present the details of variant and clinical data, including the disease outcome (lethal or nonlethal) and the severity of the disease. Odds ratios and 95% confidence intervals were used to assess the association between the type of variants (1. premature termination codon [PTC] variant in both alleles, 2. compound heterozygous with a PTC variant in one allele or splice site variant in both alleles, and 3. missense variants in both alleles or others) and clinical outcome (death or survival) using logistic regression. Statistical analysis was performed using Stata statistical software Version 16. *p* values < 0.05 were considered statistically significant.

## 3. Case Presentation

### 3.1. Clinical Data

A term female newborn was born to a healthy and nonconsanguineous couple. Her father had two healthy children from a previous marriage. She was the first gestation for her mother, who had no history of underlying disease or medication use. A prenatal ultrasound at 33 weeks of gestation showed polyhydramnios and muscular atrophy of all extremities. Neuromuscular disorders and muscular dystrophy were suspected. Amniocentesis with chromosome study was performed, revealing a normal female karyotype of 46,XX.

At birth, she developed respiratory distress and needed intubation with ventilatory support. Physical examination showed cloudy cornea, bilateral ear malformations, generalized denuded skin, and mechanically induced blistering of all extremities, including the facial area. Limb contractures and nail dystrophy were also detected (Figure 1a). EB and aplasia cutis congenita were considered in the differential diagnosis.

Figure 1Clinical presentation and sequencing data of the proband with junctional epidermolysis bullosa with pyloric atresia (JEB‐PA). (a) Generalized denuded skin and mechanically induced blistering of all extremities and facial area were observed. Cloudy cornea, bilateral ear malformation, and limb contractures were also detected. (b) Ultrasonography revealed (*A*) a large cystic lesion in mid abdomen, (*B*) renal parenchymal disease, (*C*) multiple echogenic lesions within the calyces, and (*D*) severe left hydronephrosis. The findings were consistent with ureterovesical junction (UVJ) obstruction and suspected renal papillary necrosis. (c) Sanger sequencing of proband and maternal PCR’s product spanning Exon 21 represented the heterozygous C to T transition at nucleotide Position 2533 resulting in premature termination codon, whereas the paternal sequence was normal. (d) The sequencing of proband and paternal PCR’s product spanning Exon 13 showed the heterozygous deletion of T at nucleotide Position 1614 resulting in premature termination codon, whereas the maternal sequence was normal.

**Figure:**
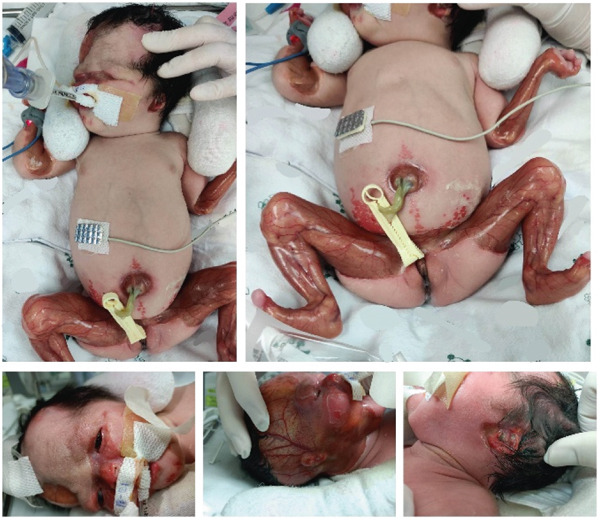
(a)

**Figure:**
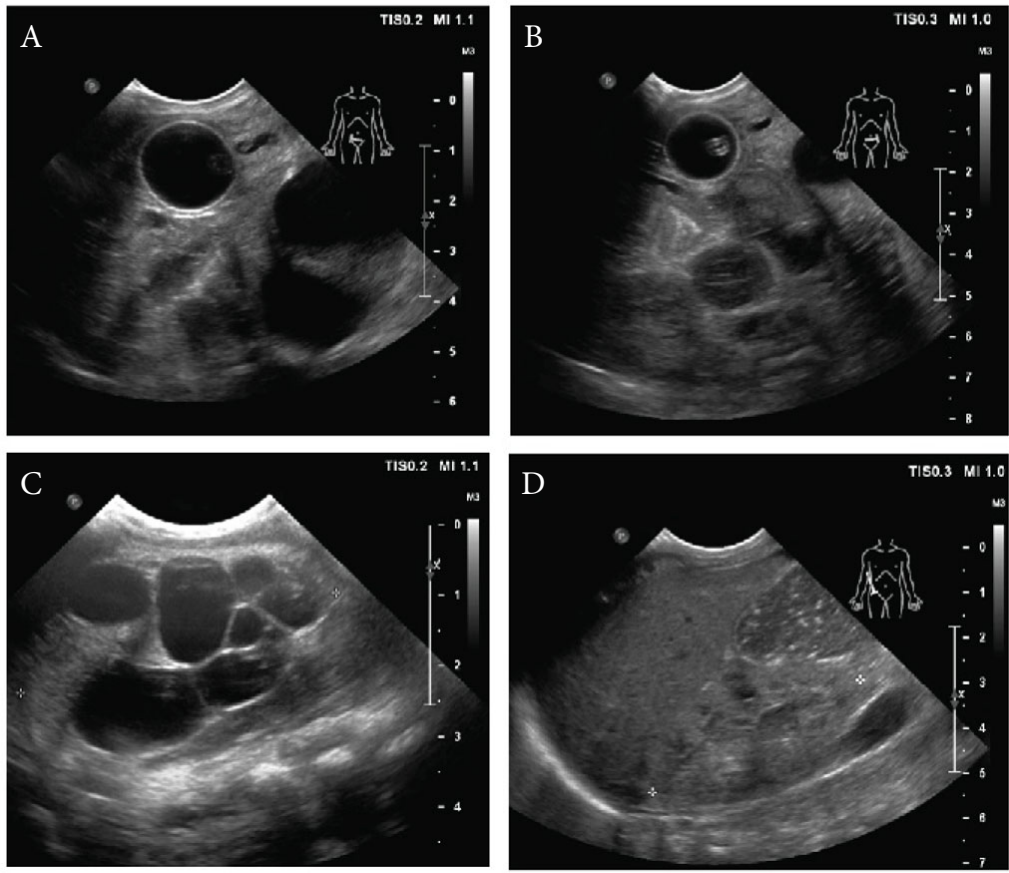
(b)

**Figure:**
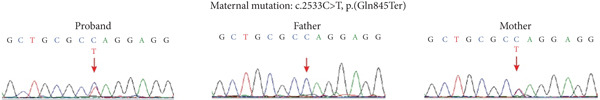
(c)

**Figure:**
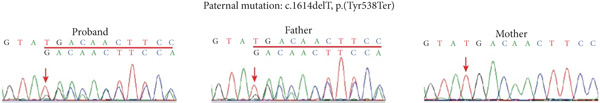
(d)

Empirical antibiotics with ampicillin and cefotaxime were administered then were escalated to acyclovir and vancomycin. Infectious causes were identified using PCR for herpes virus and immunoglobulin tests for cytomegalovirus (CMV), varicella‐zoster virus (VZV), and rubella. The results were all reported as negative. The patient had feeding intolerance. An abdominal ultrasound showed a single bubble sign and multiple dilated fluid‐filled bowel loops, indicating gastrointestinal atresia. Parenteral nutrition was introduced. The patient developed oliguria. Kidney and urinary tract ultrasound revealed renal parenchymal disease, multiple echogenic lesions within the calyces, severe left hydronephrosis, and a well‐distended urinary bladder with a small mixed solid‐cystic lesion (Figure 1b). The findings were consistent with ureterovesical junction (UVJ) obstruction and suspected renal papillary necrosis. A suprapubic cystostomy was performed by the urologist.

Due to severe loss of skin and the prolonged need for a catheter, she developed persistent sepsis with *Staphylococcus haemolyticus* and *Candida parapsilosis*. Despite our efforts, we were unable to eliminate all pathogens, resulting in an unstable clinical course. She passed away at the age of 2 months from *Pseudomonas aeruginosa* ventilator‐associated pneumonia.

### 3.2. Variant Causing Disease Identification

Trio‐WES analysis revealed compound heterozygous variants in the *ITGB4* gene. The first variant was a known nonsense variant, c.2533C>T, p.(Gln845Ter), which was inherited from the mother, confirmed by Sanger sequencing (Figure 1c). This variant also caused a PTC (in Exon 21 out of 40 exons). We classified this variant as a pathogenic variant according to ACMG/AMP guideline. It is a null variant in a gene where loss of function is a known mechanism of disease. The variant was not found in the gnomAD (https://gnomad.broadinstitute.org) and in‐house Thai Exome databases. This variant has been previously reported in *trans* with another frameshift indels variant in a female infant who was diagnosed with JEB with pyloric atresia [11]. Another variant was a 1‐bp deletion, c.1614delT, p.(Tyr538Ter), which was inherited from the father. This pathogenic variant was predicted to cause a PTC of the protein in Exon 13 out of 40 exons. The variant was not found in the gnomAD and in‐house Thai Exome databases. Sanger sequencing showed that this variant was inherited from the father (Figure 1d), confirmed to be in *trans* with the c.2533C>T variant. The c.1614delT was a novel finding.

## 4. Genotype–Phenotype Correlation of *ITGB4* Variant in JEB‐PA Cases

We have summarized 50 cases of JEB‐PA, including case information, disease severity, and variant details in Table 1. We reported the cases divided into 31 lethal cases and 19 nonlethal cases and categorized them as compound heterozygous and homozygous variants. The severity of skin involvement ranged from mild or localized blisters to extensive aplasia cutis.

**Table 1:** Clinical manifestations and *ITGB4* mutations of JEB‐PA cases.

**Case [ref.]**	**Gestation age (weeks)**	**Sex**	**Ethnic**	**Consanguinity**	**Clinical manifestations**				**Outcome**	***ITGB4* variant**					
					**Level of skin involvement**	**Nail/teeth/hair/ocular**	**Another GI tract**	**Kidney and urinary tract**		**Maternal**	**Paternal**	**Type**	**Exon**	**Domain**	**Classification**
*Lethal cases with compound heterozygous variant*															
This study	—	Female	Thai	No	Extensive	Nail dystrophy cloudy cornea	Gastrointestinal atresia	Yes	Died at 2 months	c.2533C>T, p.Gln845Ter	c.1614delT, p.Tyr538Ter	PTC/PTC	21/13	CP/EGF2	P/P
1 [12]	—	Female	French and Italian	No	Extensive + oral mucosa involvement	—	—	—	Died at 8 months	c.3793+2dup	c.1141del, p.Leu381Phefs∗56	Spl/PTC	I30/10	FnIII2/EC	LP/LP
2 [13]	—	Male	Japanese	No	Extensive	—	—	—	Died at 11 days	c.3434del, p.Phe1145Serfs ^∗^15	c.4050_4057del, p.Asp1351Serfs ^∗^15	PTC/PTC	28/32	FnIII1/CP	LP/LP
3 [14]	—	Male	Indian	NR	Extensive	—	—	—	Died at 3 weeks	c.733T>G, p.Cys245Gly	c.124_125del, p.Val42Profs ^∗^4	Mis/PTC	7/3	VWFA/PSI	LP/LP
4^a^ [14]	32 weeks	Male	Indian	NR	Extensive		—	—	Died at 3 months	c.733T>G, p.Cys245Gly	c.124_125del, p.Val42Profs ^∗^4	Mis/PTC	7/3	VWFA/PSI	LP/LP
5 [14]	34 weeks	Male	—	No	Extensive	Nail dystrophy	Esophageal atresia	—	Died at 4 days	Not found	c.217C>T, p.Gln73 ^∗^	‐/PTC	‐/4	‐/PSI	‐/P
6 [15]	—	Male	—	No	Extensive + oral mucosa involvement	—	—	—	Died at 3 months	c.4632_4633del, p.Arg1545Serfs ^∗^26	c.2214C>A, p.Cys738 ^∗^	PTC/PTC	35/18	FnIII3/TM	P/LP
7 [16]	—	Female	—	NR	Mild	—	—	—	Died at 4.5 months	c.658del, p.Leu220Cysfs ^∗^62	c.754C>T, p.Arg252Cys	PTC/Mis	7/8	VWFA/VWFA	P/LP
8 [16]		Male	—	NR	Extensive	—	Esophageal, tracheal, and small intestinal involvement	Yes	Died at 2 months	c.391G>T, p.Asp131Tyr	c.818G>A, p.Gly273Asp	Mis/Mis	5/8	VWFA/VWFA	LP/LP
9 [16]	—	Female	—	NR	Mild	—	Obstructed sigmoid colon	—	Died at 1 month	c.1874_1876delinsC, p.Leu625Profs ^∗^24	c.974T>A, p.Val325Asp	PTC/Mis	16/8	EC/VWFA	P/LP
10 [17]	—	Female	Italian	No	Extensive	—	Esophageal stenosis	—	Died at 1 day	c.953_955del, p.Asn318del	Not found	Inf del/‐	8/‐	VWFA/‐	LP/‐
11 [17]	—	Female	Turkish	No	Extensive	—	—	—	Died at 7.5 months	c.4505_4508dup, p.Thr1504Leufs ^∗^69	c.754C>T, p.Arg252Cys	PTC/Mis	34/8	CP/VWFA	LP/LP
12 [18]	—	Female	Korean	Unknown	Extensive	—	—	—	Died at 2 years	c.600dup, p.Phe201Leufs ^∗^15	c.1274A>C, p.Gln425Pro	PTC/Mis	7/11	VWFA/EC	LP/P
13 [19]	35 weeks	Female	—	No	Extensive	—	—	—	Died at 13 days	c.3807delC, p.Met1270 ^∗^	c.310del, p.Gln104Lysfs ^∗^57	PTC/PTC	31/5	FnIII2/EC	LP/P
14 [20]	30 weeks	Female	Caucasian	No	Mild	—	Gastrointestinal bleed	—	Died at 4.5 months	c.658delC, p.Leu220Cysfs ^∗^62	c.754C>T, p.Arg252Cys	PTC/Mis	7/8	VWFA/VWFA	P/LP
15 [11]	26 + 4 weeks	Female	—	No	Extensive	—	—	—	Died at 18 days	c.2533C>T, p.Gln845 ^∗^	c.600dup, p.Phe201Leufs ^∗^15	PTC/PTC	21/7	CP/VWFA	LP/P
16 [21]	36 weeks	Male	Romanian	No	Extensive	Ocular involvement	—	Yes	Died at 20 days	c.2783‐2A>G	c.470_566+182del (279 bps deletion), p.Ala157Glyfs ^∗^2	Spl/PTC	I24/6	CP/VWFA	P/P
17 [21]	35 weeks	Male	Spanish	No	Extensive + oral mucosa involvement	Onychodystrophy	Duodenal atresia	—	Died at 33 days	c.997T>G, p.Tyr333Asp	c.3321_3331del, p.Asp1109Hisfs ^∗^24	Mis/PTC	8/28	EC/CP	P/P
18^b^ [21]	34 weeks	Male	Spanish	NR	Extensive	—	—	Yes	Died at 17 days	c.3707_3725del, p.Thr1236Serfs ^∗^29	c.701G>T, p.Gly234Val	PTC/Mis	30/7	FnIII2/VWFA	P/LP
19^b^ [21]		Male	Spanish	NR	Extensive	—	Esophageal atresia	—	Died at 1 month						
20 [22]	30 weeks	Male	Taiwanese	No	Extensive	Toenail dystrophy	Gastrointestinal bleeding	Yes	Died at 3 months	c.3719G>A, p.Trp1240 ^∗^	c.121T>C, p.Cys41Arg	PTC/Mis	30/3	FnIII2/PSI	LP/P
*Lethal cases with homozygous variant*															
21 [23]	—	—	German	Yes	Extensive	—	—	—	Died at 1 month	Homozygous: c.3793+1G>A		Spl	30	FnIII2	P
22 [14]	—	Male	Pakistani	Yes	Extensive + oral mucosa involvement	—	Protein‐losing enteropathy	—	Died at 66 days	Homozygous: c.4501_4502del, p.Ser1501Thrfs ^∗^70		PTC	34	CP	LP
23 [15]	—	Female	—	NR	Extensive	—	—	—	Died at 2 weeks	Homozygous: c.182G>A, p.Cys61Tyr		Mis	4	PSI	P
24 [16]	—	Male	—	NR	Mild	—	—	—	Died at 2.5 months	Homozygous: c.5350C>T, p.Gln1784 ^∗^		PTC	40	CP	LP
25 [24]	—	—	Jordanian	Yes	Extensive	—	—	—	Died at 4 months	Homozygous: c.1378‐2A>G		Spl	I11	EGF1	LP
26 [24]	—	—	Spanish	Yes	Extensive	—	—	—	Died at 4 months	Homozygous: c.3321_3331del, p.Asp1109Hisfs ^∗^24		PTC	28	CP	P
27 [25]	—	Female	Italian	No	Extensive	—	—	—	Died at 2.5 months	Homozygous: c.175_207del, p.Arg59_Ala69del		Inf del	4	PSI	LP
28 [26]	37 weeks	Female	—	Yes	Extensive + oral mucosa involvement	—	—	—	Died at 58 days	Homozygous: c.4505_4508dup, p.Thr1504Leufs ^∗^69		PTC	34	CP	LP
29 [27]	40 weeks	Female	Turkish	Yes	Extensive	Nail dystrophy	—	—	Died at 33 days	Homozygous: c.3793+1G>A		Spl	30	FnIII2	P
30 [28]	32 weeks	Male	—	Yes	Extensive	Absence of toenails	—	—	Died at 5 days	Homozygous: c.3111+1G>A + *KRT10*: c.1498G>T, p.Gly500 ^∗^, likely pathogenic		Spl	26	Calx‐*β*	LP
*Nonlethal cases with compound heterozygous variant*															
31 [29]	35 weeks	Male	British	No	Mild	Nail dystrophy Abnormal dentition Enamel hypoplasia	—	Yes	Alive at 6 years	c.3793+1G>A	c.4643G>A, p.Trp1548 ^∗^	Spl/PTC	I30/35	FnIII2/FnIII3	P/LP
32 [29]	36 weeks	Male	British	NR	Mild	Nail dystrophy	—	Yes, since fetal	Alive at 3 years	c.112T>C, p.Cys38Arg	c.4620del, p.Thr1542Hisfs ^∗^5	Mis/PTC	3/35	PSI/FnIII3	LP/P
33 [30]	—	Male	—	No	Extensive	Nail dystrophy	—	—	Alive at 18 months	c.467T>C, p.Leu156Pro	c.1660C>T, p.Arg554 ^∗^	Mis/PTC	5/14	VWFA/EGF3	LP/P
34 [15]	—	Male	Bangladeshi	NR	Mild	—	Antral atresia	—	Alive at 10 months	c.3841C>T, p.Arg1281Trp	c.754C>T, p.Arg252Cys	Mis/Mis	31/8	FnIII2/VWFA	LP/LP
35 [31]	—	Male	—	No	Extensive	—	—	Yes	Alive at 14 years	c.3793+1G>A	c.3977‐19T>A	Spl/Spl	I30/I31	FnIII2/CP	P/LP
36 [16]	—	Female	—	NR	Extensive	—	—	—	Alive at 15 months	Not found	c.847C>T, p.Arg283Cys	‐/Mis	‐/8	‐/VWFA	‐/LP
37 [16]	—	Female	—	NR	Mild	Enamel hypoplasia Ocular involvement	Laryngeal obstruction	Yes	Alive at 13 years	c.1007T>C, p.Leu336Pro/	c.3674G>A, p.Arg1225His	Mis/Mis	9/30	EC/FnIII2	LP/LP
38 [32]	35 weeks	Male	Japanese	No	Mild	Toenail dystrophy Enamel hypoplasia and dental caries Progressive alopecia, spared eyelashes, and eyebrows	—	Yes	Alive at 12 years	c.2168C>G, p.Pro723Arg	c.1938del, p.Thr647Argfs ^∗^122	Mis/PTC	18/16	EC/EC	VUS/P
39 [20]	—	Male	Chinese	No	Mild	—	—	—	Alive at 4.5 years	c.264G>A, p.Glu88=	c.3112‐1G>A	Spl/Spl	4/27	EC/calx‐*β*	VUS/LP
40 [33]	31 weeks	Female	—	No	Mild	Onychodystrophy Enamel hypoplasia and caries	Gastroenteritis	Yes	Alive at 8 years	c.3977‐19T>A	c.3338_3354del, p.Thr1113Ilefs ^∗^18	Spl/PTC	I31/28	CP/CP	LP/LP
41 [21]	32 weeks	Female	Spanish	No	Mild + oral mucosa involvement	Onychodystrophy and toenail loss Enamel hypoplasia and caries	—	Yes	Alive at 8 years	c.997T>G, p.Tyr333Asp	c.1370G>A, p.Cys457Tyr	Mis/Mis	8/11	EC/EGF1	P/LP
42 [34]	37 weeks	Female	Japanese	No	Extensive	Onychodystrophy	—	—	Alive at 7 years	c.1274A>C, p.Gln425Pro	c.1549del, p.Glu517Serfs ^∗^252	Mis/PTC	11/13	EC/EGF2	VUS/LP
43 [35]	36 weeks	Female	—	No	Mild	—	Denudation of stomach, duodenum, and colon associated with chronic inflammation	—	Alive at 34 months	c.794dup, p.Ala266Serfs ^∗^5	c.1608C>T, p.Cys536=	PTC/Syn	8/11	VWFA/EGF2	P/VUS
44 [36]	At term	Male	—	No	Mild + oral mucosa involvement		Life‐threatening diarrhea	—	Alive at 2 months	c.3793C>T, p.Arg1265 ^∗^	c.805T>G, p.Tyr269Asp	PTC/Mis	30/8	FnIII2/VWFA	P/VUS
*Nonlethal cases with homozygous variant*															
45 [15]	—	Female	Turkish	Yes	Mild	Nail dystrophy Onychomycosis Enamel hypoplasia and caries	—	—	Alive at 7 years	Homozygous: c.1684T>C, p.Cys562Arg		Mis	14	EGF3	LP
46 [15]	—	Male	Jewish	Yes	Mild	Nail dystrophy	—	Yes	Alive at 4 months	Homozygous: c.3841C>T, p.Arg1281Trp		Mis	31	FnIII2	LP
47 [16]	—	Female	—	NR	Mild	Corneal erosions	—	—	Alive at 13 months	Homozygous: c.4631_4632del, p.Leu1544Glnfs ^∗^27		PTC	35	FnIII3	P
48 [21]	31 weeks	Female	Pakistan	Yes	Mild	Onychodystrophy Ocular involvement	—	—	Alive at 3 years	Homozygous: c.3674G > A, p.Arg1225His		Mis	30	FnIII2	LP
49 [37]	—	Male	Lebanese	Yes	Mild	Onychodystrophy Enamel hypoplasia and caries	Diarrhea and protein‐losing enteropathy	Yes	Alive at 36 years	Homozygous: c.4631_4632del, p.Leu1544Glnfs ^∗^27		PTC	35	FnIII3	P

Abbreviations: Calx‐*β*, sodium–calcium exchanger motif which contains *β*‐sheet; CP, cytoplasmic domain; EC, extracellular domain; EGF, EGF‐like domain; FnIII, Fibronectin type III‐like domain; Inf del, in‐frame deletion; Mis, missense; NR, not reported; PSI, plexin–semaphorin–integrin domain; PTC, premature termination codon; Spl, splice site variant; Syn, synonymous; TM, transmembrane domain; VWFA, von Willebrand Factor A domain.

^a^The patient is the younger brother of Case 3.

^b^Dizygotic twin.

According to the disease‐causing variants in the *ITGB4* gene, null variants predicted to induce PTC (nonsense and frameshift indels) were predominant in the lethal forms (32 out of a total of 62 alleles), whereas missense variants were most found in the nonlethal forms (18 out of a total of 38 alleles). In the lethal cases of JEB‐PA, 23 cases (74%) had PTC variants in at least 1 allele, 4 cases (13%) had homozygous splice site variants, 2 cases (6%) had homozygous missense variants, and 2 cases (6%) had in‐frame deletion variants. In contrast, in nonlethal cases, 12 cases (67%) had missense variants in at least 1 allele, and 4 cases (22%) had splice site variants. We found eight nonlethal cases with PTC variants in compound heterozygous with missense (five cases), splice site (two cases), and synonymous (one case) variants. It is important to note that homozygous PTC variants have been identified in two cases with nonlethal JEB‐PA.

**Figure 2:**
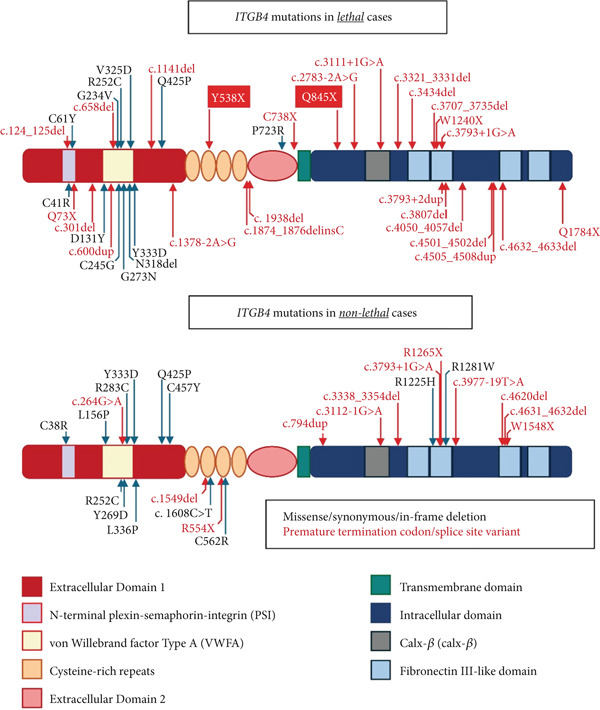
Diagram of the *ITGB4* gene and its previously reported mutations divided into two groups, missense/synonymous/in‐frame deletion and premature termination codon/splice site variant, in lethal and nonlethal cases.

The variants were predominantly found in the Fibronectin II–like domain: FnIII (26 alleles), von Willebrand factor Type A domain: VWFA (21 alleles), cytoplasmic domain: CP (17 alleles), and extracellular domain: EC (11 alleles), respectively. The pathogenic variants commonly found in the FnIII and CP domains were PTC and splice site variants, whereas missense variants were more commonly found in the VWFA domain, the ligand‐binding site (Figure 2).

The result of logistic regression showed that the presence of a PTC variant in both alleles indicated a significantly increased risk of the lethal form in JEB‐PA, with an odds ratio of 7.875 (95% CI 1.10–56.12, *p* = 0.039), compared to the group of missense variants and others. Meanwhile, compound heterozygous PTC or splice site variants in both alleles were associated with a borderline significant increased risk of the lethal form (odds ratio 3.15; 95% CI 0.74–13.45; *p* = 0.121).

## 5. Discussion

We reported a JEB‐PA patient with compound heterozygous variants in the *ITGB4* gene, consisting of a known nonsense variant (c.2533C>T) and a novel frameshift indels variant (c.1614delT). Both variants are predicted to cause PTCs, resulting in the activation of the nonsense‐mediated mRNA decay (NMD) pathway [38]. This leads to downregulation of *ITGB4* mRNA levels, resulting in the absence of functional *β*4 integrin synthesis. The histopathological examination for EB, including immunofluorescence mapping (IFM) and transmission electron microscopy (TEM), was not performed in this patient because of wide space of denuded skin and unstable clinical course.

Nevertheless, we performed trio‐WES analysis in our case, which provided the definitive diagnosis of JEB‐PA and identified the causal pathogenic variants. Although the discovery of causal pathogenic variants may not improve the survival of EB patients because EB is still an incurable disease at this time and treatments are only symptomatic, a better understanding of its pathogenesis has made it possible to develop potential therapeutic approaches for the future. Moreover, understanding the genetic background of EB‐affected families could help decrease incidence through genetic counseling, family planning, or prenatal diagnosis.

Vidal et al. first reported *ITGB4* variants causing JEB‐PA in 1995 [12]. Since then, more than 50 cases of *ITGB4*‐related JEB‐PA have been reported. To discover the genotype–phenotype correlation of *ITGB4* mutations in patients with JEB‐PA, we identified 50 cases reporting *ITGB4* variants together with clinical data, including the cases from our study. We found that the occurrence of PTC variants in both alleles was associated with severe phenotypes and lethality of JEB‐PA. However, two cases (Cases 47 and 49) carrying homozygous PTC variants were reported as nonlethal forms [16, 37]. Both cases harbor the homozygous c.4631_4632del variant located in Exon 35 of 40, which would typically be expected to cause severe phenotypes due to complete degradation of NMD. Interestingly, Nakano et al. performed IFM on a skin biopsy from a patient with the c.4631_4632del variant and detected weak but positive *β*4 integrin staining [16]. This suggests that the truncated *β*4 integrin protein resulting from this variant may retain partial function, potentially accounting for the milder clinical presentation and nonlethal phenotype observed in these cases. Therefore, this variant may represent an exception to the usual severe outcome expected from homozygous PTC mutations.

A splice site variant, a type of null variant, results in alternative donor or acceptor sites, leading to alternative splicing, including exon skipping or intron inclusion, and consequently disrupts gene production via NMD. Even though splice site variants were associated with the lethal form in most JEB‐PA patients (Cases 1, 16, 21, 25, 29, and 30), some cases had milder phenotypes and were nonlethal (Cases 31, 35, 39, and 40). We did not find an association between the location of the variant and the severity of the disease. Previous studies postulated that this phenomenon may result from tissue‐specific variation in the splicing efficiency of the *ITGB4* transcript [35]. Through IFM, they found that the splice site variant inhibited the expression of specific transcripts differently in each tissue. This resulted in the patient having minimal skin disease (where some transcripts could be detected) but severe mucocutaneous involvement (complete absence of all transcripts). The Human Protein Atlas supports this hypothesis by showing that the level of *ITGB4* RNA and protein expression varies in different tissues, even within the same organ system.

The homozygous missense variant in the *ITGB4* gene was correlated with a good clinical outcome in JEB‐PA patients. Nevertheless, it has been identified in lethal cases (Cases 8 and 23). From the case interpretation, we found that mutations in the VWFA domain were predominantly missense (Figure 2), which mostly led to the lethal form (10 out of a total of 14 variants in VWFA), either in a homozygous state (Case 8) or compound heterozygous state (Cases 3, 4, 7, 9, 11, 14, 18, and 19). The VWFA domains are found in cell adhesion and extracellular proteins containing cation‐binding sites and a ligand‐binding site, highly conserved across species [39]. This domain is a well‐studied domain involved in various important cellular functions, including basement membrane formation, cell adhesion, migration, differentiation, ligand binding, and cell signal transduction [40]. Missense mutations in this domain are predicted to significantly alter the function of *β*4 integrin and cause disease. In another lethal case with a homozygous missense variant, Case 23 carries a missense variant resulting in the substitution of cysteine located in the plexin–semaphorin–integrin (PSI) domain (Figure 2). The PSI domain is a highly conserved cysteine‐rich domain located in the extracellular part of several signaling proteins, including integrins. We found that pathogenic missense variants in the PSI domain all resulted in the substitution of cysteine (Cases 20, 23, and 32). Cysteine residues are involved in disulfide bond formation, which is important for protein structure. Taken together, missense mutations causing substitutions of highly conserved amino acids, especially in the binding site or extracellular part of integrins, might be correlated with a severe or lethal phenotype [20].

In our cohort of 50 *ITGB4*‐related JEB‐PA cases, 31 patients died. Infection was the leading cause of death, accounting for 15 cases, including sepsis resulting from skin fragility and respiratory tract infections, followed by one case each due to bleeding and multiorgan failure. The cause of death was undocumented in 14 cases. These findings highlight the substantial impact of infectious complications on patient survival and underscore the urgent need for comprehensive infection prevention strategies, early antimicrobial interventions, and multidisciplinary supportive care to improve outcomes in *ITGB4*‐related disorders.

Based on this preliminary overview of the differences in clinical phenotypes and genetic variants of *ITGB4*‐related cases across Asian and European populations, no clear distinctions were apparent in the severity of skin involvement, key clinical manifestations such as nail dystrophy and gastrointestinal atresia, or the overall variant spectrum between the two groups. Both populations appeared to show a predominance of compound heterozygous and homozygous variants, including PTCs and missense mutations affecting similar functional domains of the *ITGB4* gene. Furthermore, outcomes in lethal and nonlethal cases seemed broadly comparable across ethnicities. While these observations suggest that the clinical presentation and underlying genetic causes of *ITGB4*‐related disorders may be broadly consistent regardless of ethnicity, they are based on limited and heterogeneous case reports. Therefore, further systematic studies with larger, well‐characterized cohorts are needed to confirm these preliminary impressions and to explore potential population‐specific differences in more detail.

This evidence illustrates the heterogeneity in clinical and genetic aspects of JEB‐PA, as well as genotype–phenotype correlations. Mutations in the *ITGB4* gene result in various presentations and degrees of severity in JEB‐PA. Interestingly, these mutations may also cause EB without pyloric atresia in certain cases of JEB [41]. Moreover, *ITGB4* variants have been associated with pyloric atresia, even in individuals who do not exhibit symptoms of EB [42]. These data reveal that there are gaps in knowledge remaining to be explored regarding the correlation of clinical, genetic, and molecular mechanisms modulating the disease. A better understanding of JEB‐PA, as well as EB, is important for the development of potential treatments in the future.

## 6. Conclusion

In summary, we have presented a rare Thai case of JEB‐PA characterized by severe skin blistering and erosion, ocular and gastrointestinal atresia, and significant kidney and urinary tract involvement, associated with compound heterozygous null variants in the *ITGB4* gene. Ultimately, identifying causative variants in JEB‐PA, as well as in EB, is crucial not only for patients and their families for diagnosis and surveillance programs but also for expanding our understanding of *ITGB4*, potentially leading to future therapeutic approaches.

## Disclosure

All authors have read and approved the final manuscript.

## Conflicts of Interest

The authors declare no conflicts of interest.

## Author Contributions

J.K. and M.T. contributed to conceptualization, data curation, methodology, software, formal analysis, software, data validation and investigation, visualization, and writing—original draft, reviewing, and editing. M.P. contributed to resources, data curation, validation, reviewing, and editing. P.T. involved in experimental investigation of the study, resources, data curation, software, and formal analysis. N.M., R.K., A.P., and P.S. were involved in resources. C.P. contributed to conceptualization and supervision. J.K. and M.T. contributed to funding acquisition.

## Funding

This study was supported by the Faculty of Medicine, Chiang Mai University (10.13039/501100010731, 76‐2565), the Chiang Mai University (10.13039/501100002842), and the CMU Junior Research Fellowship Program.

## Data Availability

The data that support the findings of this study are available on request from the corresponding author (M.T.). The data are not publicly available due to privacy or ethical restrictions.
